# Resistance Training to Muscle Failure with Variable Load Intensities: Implications for Post-Exercise Blood Pressure and Heart Rate Variability in Trained Men

**DOI:** 10.3390/jcm13082296

**Published:** 2024-04-16

**Authors:** Ali Kamil Güngör, Hüseyin Topçu, Monira I. Aldhahi, Sameer Badri Al-Mhanna, Mehmet Gülü

**Affiliations:** 1 Department of Coaching Education, Faculty of Sport Sciences, Bursa Uludağ University, 16059 Bursa, Türkiye; alikamilgungor@uludag.edu.tr; 2 Department of Physical Education and Sport, Faculty of Sport Sciences, Bursa Uludağ University, 16059 Bursa, Türkiye; huseyintopcu@uludag.edu.tr; 3 Department of Rehabilitation Sciences, College of Health and Rehabilitation Sciences, Princess Nourah bint Abdulrahman University, P.O. Box 84428, Riyadh 11671, Saudi Arabia; 4 Department of Physiology, School of Medical Sciences, Universiti Sains Malaysia, Kubang Kerian 16150, Kelantan, Malaysia; sameerbadri9@gmail.com; 5 Department of Sports Management, Faculty of Sport Sciences, Kirikkale University, 71450 Kirikkale, Türkiye

**Keywords:** resistance exercise, cardiac autonomic modulation, sympathovagal balance, tension, recovery

## Abstract

**Background**: The modulation of cardiac sympathovagal balance alters following acute resistance training (RT) sessions. Nevertheless, the precise influence of RT at varying load intensities on this physiological response remains to be fully elucidated. Therefore, the aim of this study was to compare the time course of recovery following low- (40%), moderate- (60%), and high- (80%) load-intensity RT protocols performed up to muscle repetition failure in resistance-trained men. **Method**: Sixteen young, resistance-trained men (mean age: 21.6 ± 2.5 years, mean height: 175.7 ± 8.9 cm, mean weight: 77.1 ± 11.3 kg) participated in a randomized crossover experimental design involving three sessions, each taken to the point of muscle failure. These sessions were characterized by different load intensities: low (40% of 1-repetition maximum, 1RM), moderate (60% of 1RM), and high (80% of 1RM). The exercise regimen comprised four exercises—back squat (BS), bench press (BnP), barbell row (BR), and shoulder press (SP)—with each exercise consisting of three sets. Throughout each session, heart rate variability (HRV) and blood pressure (BP) parameters were assessed both pre-exercise and during a 40 min post-exercise period, segmented into 10 min intervals for stabilization. Statistical analysis involved the use of a repeated measures ANOVA. **Results**: It was observed that the 40% and 60% RT sessions resulted in a significantly higher root mean square of successive R-R intervals (RMSSD) value compared to the 80% RT session in the post-exercise recovery process in 30 min (respectively, *p* = 0.025; *p* = 0.028) and 40 min (respectively, *p* = 0.031; *p* = 0.046), while the 40% and 60% RT sessions produced similar responses. The 40% RT session was significantly higher in the high frequency (HF) value post-exercise in 40 min compared to the 80% RT session (*p* = 0.045). **Conclusions**: Our findings suggest that engaging in resistance training (RT) sessions to muscle failure at an intensity of 80% induces acute increases in sympathetic activity, potentially leading to elevated cardiovascular stress. For individuals with normal blood pressure, it is advisable to opt for lighter loads and higher repetition volumes when prescribing RT, as heavier-load RT may carry an increased risk of cardiac-related factors.

## 1. Introduction

Resistance training (RT) is considered an important exercise method applied to increase the performance of practitioners and to improve various metabolic, musculoskeletal, and cardiovascular statuses [1]. Manipulating RT variables (exercise type, load, set and repetition, rest, volume, etc.) triggers the exercise stimulus and alters the magnitude and type of RT-induced physiological responses and adaptations [2]. RT has been associated with acute decreases in blood pressure (BP) after exercise. This status is named post-exercise hypotension, and it may play an important role in reducing chronic BP and cardiovascular risk. A slight decrease in BP (e.g., 3 mmHg) reduces the likelihood of stroke and coronary artery disease in normotensive or hypertensive individuals [3]. Therefore, RT can be a useful tool for moderating BP levels.

Accurate methods for measuring cardiac activity variables and assessing recovery during or after RT are of paramount importance. One such measurement method is heart rate variability (HRV), which reflects the fluctuations in time intervals between consecutive heartbeats and serves as a noninvasive indicator of cardiac autonomic regulation [4]. HRV analysis is conducted utilizing dedicated software, encompassing both time and frequency domains. Specifically, within the time domain, the root mean square of successive differences (RMSSD) is computed. In the frequency domain, it is used to determine the spectral power within the low-frequency (LF) and high-frequency (HF) bands presented in normalized units. Among these parameters, the RMSSD and HF reflect vagal activity, whereas LF is indicative of predominantly sympathetic activity, albeit also influenced by vagal tone to a lesser degree [4]. Previous research has demonstrated that both load intensities and training volumes can influence cardiac vagal control following an RT session [5,6]. In recent years, RT programs emphasizing muscle repetition failure for strength and hypertrophic gains have gained popularity. It has been suggested that regardless of the load intensity, similar gains in strength and hypertrophy can be achieved if exercises are performed to the point of muscle failure [7,8,9]. However, there is a paucity of studies investigating the impact of the load intensity on post-exercise cardiac autonomic modulation [10,11,12,13,14]. Given that the load intensity of RT can affect the mechanical and metabolic responses within the vasculature and subsequently influence reflex mechanisms of cardiac autonomic modulation, it is plausible that varying intensities of RT may elicit diverse responses in post-exercise cardiac autonomic modulation [10]. Understanding how RT load intensity impacts cardiac autonomic modulation is crucial for practitioners, as RT can lead to a reduction in cardiac vagal modulation for up to 12–48 h, and RT variables can modulate the magnitude and duration of this acute response [15,16].

Rezk et al. [17] examined the impact of two distinct load intensity protocols, one involving 20 repetitions with 40% of the one-repetition maximum (1RM) and the other consisting of 10 repetitions with 80% of the 1RM, on BP and HRV in both untrained men and women. Their findings indicated similar results in HRV parameters between the two protocols. However, they observed a more significant decrease in diastolic blood pressure (DBP) at 15 and 30 min intervals post-exercise in the 40% protocol compared to the 80% protocol. No significant differences were noted in systolic blood pressure (SBP). A study involving hypertensive postmenopausal women compared the 6-repetition maximum (RM) and 15RM RT protocols. This study reported greater changes in HRV markers after the 15RM session than after the 6RM session [14]. Although the 6RM session exhibited higher SBP compared to the control session, there were no significant differences in DBP. The 6RM session was higher than the control session in SBP but not in DBP. In contrast, another study showed greater alterations of HRV parameters during 60 min (min.) after exercise in 70% load intensity (up to muscle failure) sessions compared to 50% load intensity [10]. RT performed until muscle failure at different intensities may produce different responses in HRV and BP.

The existing body of literature lacks a consensus on how the RT load intensity affects post-exercise BP and HRV. Ascertaining the cardiovascular repercussions associated with RT sessions conducted at different load intensities is an ongoing area of research that can offer valuable insights for fitness professionals when designing RT programs. To the best of our knowledge, there have been no prior studies examining the impact of low (40%), moderate (60%), and high (80%) RT load intensities, all performed until muscle failure using barbell-based exercises targeting major muscle groups, on HRV and BP responses in physically trained men. It was suggested that RT characterized by greater muscle mass leads to elevated lactatemia [18], and therefore glycolytic involvement is associated with cardiac vagal withdrawal [6,19].

The purpose of this study was to analyze and compare the effects of RT sessions performed until muscle failure at varying load intensities (40%, 60%, and 80%) on post-exercise BP parameters and autonomic cardiac modulation in physically trained men. We hypothesized that each RT session would induce a sympathovagal imbalance; however, we anticipated that the 80% RT session would result in a more substantial and prolonged response in this regard.

## 2. Materials and Methods

### 2.1. Experimental Design

During the first two visits, the researchers determined the 1RM and conducted a retest of the 1RM measurement. In the subsequent three visits, the participants engaged in resistance training exercises until reaching the point of muscle failure. These resistance training sessions were conducted at load intensities of 40%, 60%, and 80% of their previously established 1RM. The order of these three training intensities was randomized to eliminate any potential order effects. During all three methods of resistance training (40%, 60%, and 80% 1RM), participants performed a set of exercises including back squats (BSs), bench press (BnP), barbell rows (BRs), and shoulder press (SP). This study collected data on HRV, including the RMSSD, HF, and LF components, as well as BP, including SBP and DBP. These measurements were taken at various time points, including before the exercise (pre), immediately after the exercise (post), and at 10 min intervals following the exercise for a total duration of 40 min. This study employed a randomized crossover design to ensure that each participant experienced all three load intensities, minimizing the influence of the order of the interventions on the study outcomes.

### 2.2. Subjects

Sixteen normotensive young resistance-trained men with at least 12 months of experience in RT voluntarily participated in this study. The training experience of the subjects ranged from 1 to 4 years (3/4 days per week, each training session between 60 and 75 min excluding warm-up). Subjects’ characteristics were as follows: age 21.6 ± 2.5 yr; height 175.7 ± 8.9 m; weight 77.1 ± 11.3 kg; training experience 2.4 ± 0.7; and body mass index (BMI) 24.9 ± 2.8 kg m^−2^ (Tanita Model BF-350; Tanita Corp., Tokyo, Japan). A priori power analysis was determined using the G Power software (version 3.1.9.7) for the F test family (repeated measures ANOVA, within factors) to calculate the required sample size. Assuming statistical power = β 0.80, error probability = α 0.05, and effect size ES = f 0.30 with three sessions and six measurements for a sample size was calculated as n = 15. The exclusion criteria were as follows: (a) no joint or bone injury in the last six months, (b) the absence of musculoskeletal or cardiovascular problems that might influence the performance, and (c) not using drugs or similar substances (stimulants) that affect the cardiovascular system. Also, those with a resting SBP of ≥140 mmHg and a DBP of ≥90 mmHg were excluded. Before obtaining written informed consent from the participants, they were thoroughly briefed on the research procedures, requirements, potential benefits, and any associated risks. All participants willingly signed an informed consent form, indicating their understanding and agreement to participate in the study. This study was conducted in accordance with the Declaration of Helsinki and was approved by the local Clinical Research Ethics Committee and, as such, conformed to the code (2022-9/11) of ethics of the World Medical Association.

### 2.3. Procedures

#### 2.3.1. One-Repetition Maximum Test (1RM)

Prior to conducting the 1RM test, a researcher provided a demonstration of the correct form and technique for each exercise. This demonstration was aimed at preventing any practice errors that might occur during the test. The initial load for each 1RM test was estimated based on the typical weight that participants commonly used in their regular RT sessions. In cases where a subject was unable to lift the 1RM weight or, conversely, lifted more than their 1RM on the first attempt, the weight was adjusted, either increased or decreased, by 5–10 kg as needed. There was a 3 min rest period provided between each attempt during the 1RM test, and a total of three attempts were permitted per test session. The 1RM testing sequence began with the back squat (BS: 123.2 ± 21.8), followed by the bench press (BnP: 96.4 ± 13.5), barbell rows (BR: 93.9 ± 20.2), and shoulder press (SP: 62.5 ± 10.5) exercises, with a standardized 5 min rest interval between each exercise. Verbal encouragement was provided to the participants during the tests to motivate their best performance. Importantly, all the 1RM tests were conducted on the same day. Once the maximum load for each participant had been determined through these tests, the loads corresponding to 40%, 60%, and 80% of their 1RM were calculated and used for subsequent resistance training sessions.

#### 2.3.2. Training Interventions

A total of 72 h after the 1RM test, subjects performed three different RT protocols across three sessions in a randomized order. Before the warm-up session, HRV was recorded in the supine position on the mat for 10 min, and BP was measured immediately afterward. As a warm-up routine, subjects performed 2 min jogging before exercise, followed by 1 × 15 back squats and 1 × 15 bench presses at 40% of their 1RM. After completing the warm-up routine, a period of 120 s of rest was given prior to the start of the exercises. In each exercise session, subjects practiced exercises BS, BnP, BR, and SP, respectively. Exercises were performed in 3 sets, with 2 min of rest between sets and 3 min of rest between exercises. Each set was completed until muscle failure. Muscle failure was described as the inability to complete a repetition mechanically and the subject choosing to stop due to the perception of being unable to continue the exercise [20]. The repetitions were made as two seconds eccentric and fast concentric. At the end of each set, subjects’ ratings of the perceived exertion levels (RPEs), number of repetitions, and set load were recorded. HRV was recorded during exercise, and BP was measured immediately after each exercise session. After the exercise, HRV was recorded at 10 min intervals for 40 min, and BP measurements were made at the end of each 10 min. None of the subjects reported any adverse events after the exercise sessions.

#### 2.3.3. Rating of Perceived Exertion Assessment

The RPE of subjects’ exercise tolerance, such as strain, pain, and fatigue, was measured using the Borg scale (from no effort 6 to maximum effort 20) [21]. The Borg scale was as follows: extremely light (7–8), very light (9–10), light (11–12), somewhat hard (13–14), hard (15–16), very hard (17–18), and extremely hard (19). During the exercises, the RPE expressed by the subjects at the end of each set was recorded.

#### 2.3.4. Blood Pressure Assessment

SBP and DBP were measured using an automated oscillometric device (Omron M2 HEM-7121-E, Kyoto, Japan). The equipment was automatically calibrated before each use. Measurements were made following the recommendations of the American Heart Association [22]. Before each exercise session, subjects lie on a rubber mat in the supine position for a period of 10 min to determine resting BP. After the exercise session, the subjects lay in a supine position for 40 min in a quiet room. BP measurements were taken at specific time intervals, including immediately post-exercise (0–5 min) and at subsequent intervals of 10, 20, 30, and 40 min following the exercise session. In total, six measurements were taken during this post-exercise period to comprehensively assess the cardiovascular response.

#### 2.3.5. Heart Rate Variability Assessment

**Figure 1:**
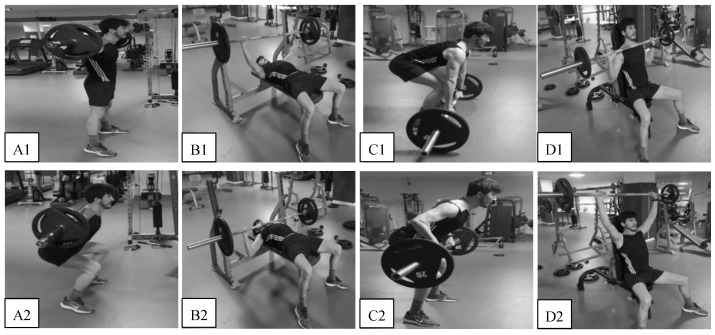
Resistance training. (**A1**,**A2**) Back squat, (**B1**,**B2**) bench press, (**C1**,**C2**) barbell row, and (**D1**,**D2**) shoulder press.

The measurement of consecutive R-R intervals of the heart rate was recorded at 10 min pre-, post-, and 40 min post-exercise with 10 min intervals using an HR monitor (Polar V800, Polar Electro OY, Kempele, Finland). The data were recorded on the equipment and then immediately transferred to the computer via the Polar Flow application for analysis. Kubios software version 3.5.0 (Biosignal Analysis and Medical Imaging Group, Department of Physics, University of Kuopio, Kuopio, Finland) was used to calculate HRV parameters in the time and frequency domains. The software used automatically eliminated the noise. Artifact correction was set to a medium correction threshold, and it never passed over 2% of the signal (beats corrected). Fast Fourier transformation was selected for the spectral analysis in the frequency domain. For HRV analysis in the frequency domain, we used the spectral components of LF (0.04–0.15 Hz) and HF (0.15–0.4 Hz) in normalized units. Normalized unit HF (HFnu) was used as an indicator of cardiac parasympathetic activity, whereas normalized unit LF (LFnu) was used by both sympathetic and parasympathetic modulations [4]. One of the time domains used to assess cardiac modulation was the RMSSD. This metric is commonly referred to as a parasympathetic cardiac modulation measure. The data analysis was performed for the last 5 min of the period since epochs of 5 min are recommended when taking short-term recordings [4]. The data samples were as follows: pre-10: 5–10 min and post: 0–5 min; 10: 5–10 min; 20: 15–20 min; 30: 25–30 min; and 40: 35–40 min. Considering circadian rhythm, all sessions were performed between 9.00 and 12.00 a.m. HRV data were collected with subjects in a supine position in a quiet room with temperatures maintained between 20 and 24 °C. The RT is presented in Figure 1.

### 2.4. Statistical Analysis

The data were analyzed using SPSS version 28.0 (IBM Corp., Armonk, NY, USA). Descriptive parameters were given as the mean and standard deviation. The normality of all variables was analyzed with the Shapiro–Wilk test. To define the reliability of the 1RM test, intraclass correlation coefficients (ICCs) with single-measure ICCs were determined for BS, BnP, BR, and SP. The classification of ICCs was defined as poor reliability (ICC ≤ 0.40), moderate reliability (ICC > 0.41 ≤ 0.75), and excellent reliability (ICC > 0.75). The total repetition and training volume of sessions were compared using the one-way ANOVA test. A two-way repeated measures ANOVA (3 sessions × 6 times) was applied to evaluate the effect and interaction between session and time in BP and HRV parameters. Multiple comparisons were executed using Bonferroni adjustment. The significance level was set at *p* < 0.05. The partial eta-squared ($n_{p}^{2}$) of repeated measures ANOVA and Cohen’s d for pairwise differences were used to calculate the ES. The magnitudes of the ES were classified as trivial (0.0–0.2), small (0.2–0.6), moderate (0.6–1.2), large (1.2–2.0), and very large (>2.0) [23]. Pearson’s product-moment correlation coefficients (r) were used to assess the association between total repetitions and volume load with HRV variables (RMSSD, HF, and LF) post resistance training (RT) session and recovery. The associations among outcome variables were evaluated using Pearson’s product-moment correlation coefficients, categorized as follows: small (0 to 0.30), moderate (0.31 to 0.49), large (0.50 to 0.69), very large (0.70 to 0.89), and almost perfect (0.90 to 1.00). A post hoc power analysis was calculated using the G Power software (version 3.1.9.7) for the F test family (repeated measures ANOVA, within factors) to define the number of subjects. Statistical power (1-b) with three sessions and six measurements for a sample size of 16, a correlation among repeated measures of 0.5, and effect size (f = 0.25) was 0.72.

## 3. Results

The 1RM test and retest ICC was BS = 0.96, BnP = 0.94, BR = 0.94, and SP = 0.98. Significant differences were detected between sessions for total repetitions (F_2,26_ = 352.744 *p* < 0.001, $n_{p}^{2}$ = 0.964) and training volume (F_2,26_ = 45.886 *p* < 0.001, $n_{p}^{2}$ = 0.779) but not in the RPE (F_2,22_ = 1.518 *p* > 0.242, $n_{p}^{2}$ = 0.121). A significantly greater total repetition was found in 40% (*p* < 0.001) compared to 60% and 80% in post hoc pairwise comparisons. Also, a greater total repetition was detected in the 60% (*p* < 0.001) session compared to the 80% sessions. A greater training volume was found in the 40% (*p* < 0.001) and 60% (*p* < 0.001) sessions compared to the 80% sessions (Table 1).

### 3.1. BP Data

**Figure 2:**
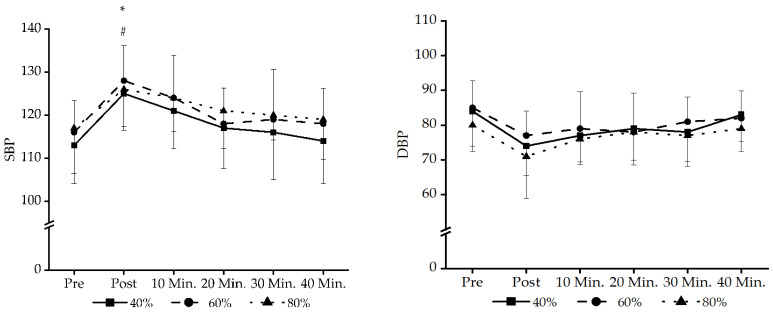
Shows the values of SBP and DBP. SBP, systolic blood pressure; DBP, diastolic blood pressure. * There is a significant difference in the 40% exercise method compared to pre-exercise *p* < 0.05. # There is a significant difference in the 60% exercise method compared to pre-exercise *p* < 0.05.

There was a main effect for the time in SBP (F_5,65_ = 10.661 *p* < 0.001, $n_{p}^{2}$ = 0.451) and DBP (F_5,65_ = 6.62 *p* < 0.001, $n_{p}^{2}$ = 0.318). The main effect of time showed that the period of the post (*p* < 0.001) and 10 min (*p* < 0.008) were lower than the pre-values in SBP. The main effect of time showed that the period of the post (*p* < 0.001) and 10 min (*p* < 0.005) were lower than the pre-values in DBP. There was no main effect for sessions in SBP (F_2,26_ = 2.036 *p* < 0.151, $n_{p}^{2}$ = 0.016) and DBP (F_2,26_ = 2.742 *p* < 0.083, $n_{p}^{2}$ = 0.174). Additionally, an interaction between session and time was not detected in SBP (F_10,130_ = 0.2014 *p* < 0.995, $n_{p}^{2}$ = 0.016) and DBP (F_10,130_ = 0.237 *p* < 0.992, $n_{p}^{2}$ = 0.018) (Figure 2).

### 3.2. HRV Data

No main effects for sessions were detected in the RMSSD (F_2,26_ = 1.194 *p* < 0.319, $n_{p}^{2}\;$= 0.084), HF (F_2,26_ = 0.739 *p* < 0.487, $n_{p}^{2}$ = 0.054), and LF (F_2,26_ = 1.098 *p* < 0.349, $n_{p}^{2}\;$= 0.078). There were no significant interactions between times and sessions in the RMSSD (F_10,130_ = 1.264 *p* < 0.257, $n_{p}^{2}\;$= 0.089), HF (F_10,130_ = 1.912 *p* < 0.120, $n_{p}^{2}\;$= 0.128), and LF (F_10,130_ = 0.537 *p* < 0.861, $n_{p}^{2}\;$= 0.040). However, significant main effects for time were found in the RMSSD (F_5,65_ = 92.247 *p* < 0.001, $n_{p}^{2}\;$= 0.876), HF (F_5,65_ = 47.422 *p* < 0.001, $n_{p}^{2}\;$= 0.785), and LF (F_5,65_ = 52.477 *p* < 0.001, $n_{p}^{2}$ = 0.801). A post hoc pairwise comparison for the main effect of time in the RMSSD showed that the period of the post (*p* < 0.001), 10 min (*p* < 0.001), 20 min (*p* < 0.001), 30 min (*p* < 0.001), and 40 min (*p* < 0.001) were lower than the pre-value. HF showed that the period of the post (*p* < 0.001), 10 min (*p* < 0.001), 20 min (*p* < 0.001), 30 min (*p* < 0.001), and 40 min (*p* < 0.001) were lower than the pre-value. Also, LF showed that the period of the post (*p* < 0.001), 10 min (*p* < 0.001), 20 min (*p* < 0.001), 30 min (*p* < 0.001), and 40 min (*p* < 0.001) were lower than the pre-value.

**Figure 3:**
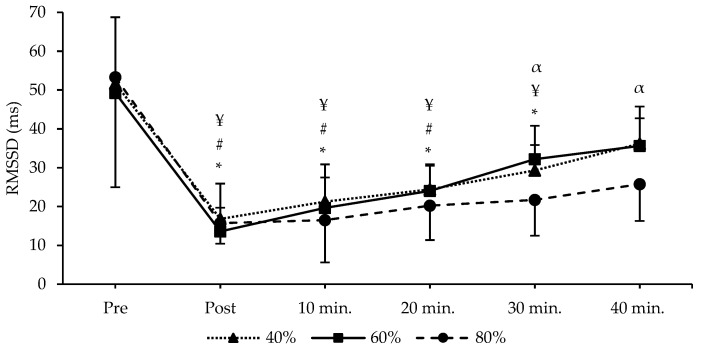
Shows the time and group interactions of the RMSSD. RMSSD, root mean of the square of the difference in the RR intervals. * There is a significant difference in the 40% exercise method compared to pre-exercise *p* < 0.05. # There is a significant difference in the 60% exercise method compared to pre-exercise *p* < 0.05. ¥ There is a significant difference in the 80% exercise method compared to pre-exercise *p* < 0.05. α There are significant differences between the 40% and 80% exercise method in 30 min and 40 min.

**Figure 4:**
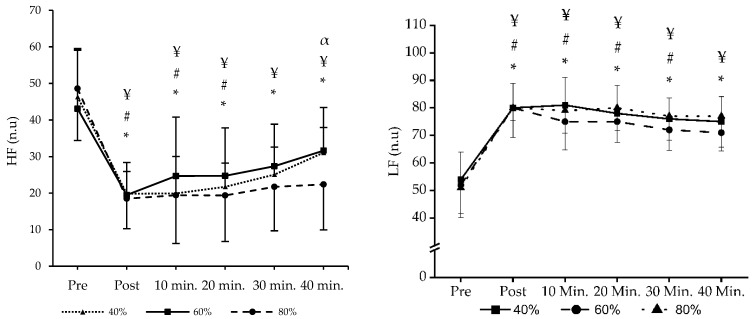
Shows the time and group interactions of HF and LF. HF, high frequency; LF, low frequency. * There is a significant difference in the 40% exercise method compared to pre-exercise *p* < 0.05. # There is a significant difference in the 60% exercise method compared to pre-exercise *p* < 0.05. ¥ There is a significant difference in the 80% exercise method compared to pre-exercise *p* < 0.05. α There are significant differences between the 40% and 80% exercise method in 40 min.

A pairwise comparison of time interactions for the RMSSD showed that the period of the post, 10, 20, and 30 min were lower than the pre-value in 40% and 80% RT sessions. However, the 60% RT session was lower than the pre-value until 20 min in the RMSSD. Additionally, the RMSSD of the 80% RT session was significantly lower at 30 and 40 min compared to the 40% and 60% RT sessions (Figure 3). For HF, all intervals were found to be lower than the pre-value in the 40% and 80% RT sessions. However, the 60% RT session was lower than the pre-value until 20 min in HF. Additionally, the HF of the 80% RT session was significantly lower at 40 min compared to the 40% RT session (Figure 4). For LF, all intervals were found to be lower than the pre-value in the 40% and 80% RT sessions. However, the 60% RT session was lower than the pre-value until 30 min in LF (Figure 4). All data HRV and BP values are shown in Table 2.

In examining the relationship between total repetitions and volume load with HRV variables (RMSSD, HF, and LF) post resistance training (RT) session and recovery, a significant negative large correlation was observed between total repetitions and the RMSSD post-exercise in sessions conducted at 60% intensity (r = −0.577, *p* = 0.031) (Table 3).

## 4. Discussion

The purpose of this study was to examine the effects of three different load -intensity RT protocols up to muscle repetition failure on the BP and HRV variables after exercise. The main findings of this study showed no significant difference in the BP variables in the main effect of session, time, and session x time. The 40% and 60% RT sessions yielded significantly higher values of the RMSSD (root mean square of successive R-R intervals) after exercise at the 30 -min and 40 -min marks compared to the 80% RT session. In terms of HRV, the 40% RT session displayed significantly higher HF values after exercise at the 40 -min mark in comparison to the 80% RT session. Interestingly, all three RT sessions exhibited similar responses in LF after exercise.

In the present study, there was no significant difference in the BP variables (SBP and DBP) between protocols. However, previous studies have reported conflicting results [11,13,14,17,24]. In a randomized crossover study on 15 hypertensive, post-menopausal women consisting of 15RM, 6RM, and control protocols, a greater increase in SBP im-mediately after exercise was found in the 6RM exercise compared to the control group. Similar responses were detected between protocols in DBP [14]. In another study, similar results were reported between the protocols in SBP and DBP after exercises with the equated intensity of 1RM 40% (18 reps.), 60% (12 reps.), and 80% (9 reps.) [13]. Rezk et al. [17] examined the impact of two different load intensities (20 reps. with 40% of 1RM versus 10 reps. with 80% of 1RM) on the BP of untrained men and women. A greater decrease in the 40% protocol than 80% was detected in DBP at 15- and 30 -min post-exercise. There were no differences between protocols in SBP. It was reported that the change in BP values after acute exercise is similar to the change in resting conditions after 8 weeks of chronic exercise [12]. Unlike other studies, the subjects in our study were trained (>12 months, at least three or four sessions weekly) and familiar with all training protocols. Acute training’s impact on BP may have been reduced as participants adapt to regular exercise. Also, training manipulations such as the training type (aerobic, resistance, etc.), load intensity (40%, 70%, etc.), rest intervals (1 min, 2 min, etc.), training status (trained or untrained), age (young or older), and subject characteristics (normotensive, prehypertensive, etc.) seem to affect the magnitude and duration of the BP after RT.

The utilization of HRV parameters as a reliable indicator of cardiovascular risk has been established. Nevertheless, the association between HRV and RT in normotensive individuals remains inadequately investigated in the existing literature. Some studies have reported similar results regarding HRV parameters after RT was conducted at various intensities [11,13,17], while a few studies presented conflicting findings [10,14]. In a study with 15 untrained young men in a crossover design in three different protocols (control, 50%, and 70% of 1-RM groups), the R-R interval and HF remained reduced during 60 min after exercise performed until muscle repetition failure at 70% intensity compared to 50% intensity, while LF remained elevated [10]. On the contrary, in another study, a lighter load and higher repetitions (15RM) demonstrated greater alterations in cardiovascular variables, including the RMSSD, HF, and LF components, immediately after exercise but not up to 60 min, compared to 6RM loads and the control group [14]. In the present study, the RMSSD (in 30 and 40 min) and HF (in 40 min) remained reduced after the exercise at 80% compared to the 40% sessions. LF showed similar responses between RT sessions. In the current study, it was observed that in the exercise performed with 40% RT intensity, the subjects ended the set before reaching the point of muscle repetition failure in most sets because they felt muscle pain after too many repetitions. In addition, the RPE values were similar in all sessions. It is stated that in low-intensity protocols with more repetitions, such as muscle failure, the time under tension will be longer, which may lead to greater training intensity [25]. However, in our study, although the number of repetitions in high-intensity exercise performed until muscle fatigue was less than in low-intensity exercise, more weight was lifted per unit time. We consider that the similar findings in the RPE are due to this. Since lower repetitions and higher load were performed at 80% RT, subjects were expected to perform repetitions close to muscle repetition failure. Researchers suggested that RT close to muscle repetition failure results in greater and longer-lasting cardiac sympathetic activation [10]. Heavier-load RT results in heightened sympathetic activation caused by a greater mechanical overload on the vascular system, leading to a subsequent reduction in HRV parameters [26]. This response may be attributed to elevated mechanical stress on the vascular system during higher-load exercise, leading to heightened mechanoreceptor activation and increased metaboreflex reaction due to reduced blood flow [19]. A plausible explanation suggests the pronounced reduction in plasma volume after higher-load RT, potentially caused by blood leakage into the interstitial space. This process may lead to a decrease in venous return, following deactivating the cardiopulmonary receptor and thus eliciting an increase in heart rate [10].

In light of the results obtained in our study, it may be recommended that practitioners consider resistance exercise with lighter loads until muscle failure as an option when designing RT programs. These findings hold particular significance for practical applications in RT protocols aimed at enhancing muscle strength or promoting hypertrophy. Previous research has suggested that when exercises are performed to the point of muscle repetition failure, there is a similarity in the strength and hypertrophic gains achieved, regardless of whether a higher or lower number of repetitions is employed [8,9,27]. By incorporating lighter loads and higher repetition volumes, practitioners can potentially mitigate cardiovascular stress associated with heavier-load RT, offering a safer and more effective approach to achieve strength and hypertrophy goals in RT programs.

It is important to acknowledge and address the limitations of this study. Blood lactate concentration and respiratory frequencies of the subjects were not controlled during the study. These variables can potentially influence cardiovascular responses to exercise and should be considered in future research to provide a more comprehensive analysis. Thus, HRV measurements were conducted in the supine position, both at rest and after exercise. It should be noted that different body positions, such as standing or sitting, can have an impact on HRV and BP variables, as indicated in previous research [28]. Future studies could explore the HRV and BP responses in various positions to better understand their influence. Lastly, the study exclusively involved normotensive young, trained subjects. Consequently, caution should be exercised when attempting to generalize the results to other populations, such as individuals with prehypertension, hypertension, females, or sedentary individuals. Further research is warranted to investigate these distinct demographic groups and assess potential variations in responses.

## 5. Conclusions

Similar increases in sympathetic activity were observed in all protocols during the exercises. However, during the post-exercise recovery period, exercise at 80% intensity took longer to reach sympathovagal balance than exercise at 40% intensity. This may lead to a risk of prolonged exposure to cardiovascular stress after exercise performed at high intensities until muscle failure. Conversely, the 40% and 60% RT sessions exhibited similar cardiac activity responses during the post-exercise recovery period. Additionally, there were no significant differences in the BP parameters observed between the sessions. Based on these findings, we recommend that normotensive individuals consider using lighter loads and incorporating higher repetition volumes when designing their RT programs. This approach may help mitigate the potential cardiac risk factors associated with heavier-load RT. However, it is essential to balance these considerations with exercise economy and user comfort when prescribing RT. For future research, it is crucial to validate these results in diverse populations and investigate the long-term effects of various RT protocols. This will provide a more comprehensive understanding of the risk-to-benefit ratio associated with different RT approaches and their implications for cardiovascular health.

## Figures and Tables

**Table 1:** Comparison of total repetitions, training volume, set durations, and RPE values *.

Exercises	Total Repetitions (1)	Volume Load (kg) (2)	Total Set Duration (3)	RPE (Median)	Pairwise Comparisons	*p*/Cohen’s *d*
40% (a)	271.7 (30.7) †	10,726.1 (2346.5)	1048.54 (64.3) †	16 (16 ± 1)	a1–b1	0.001/3.815
					a1–c1	0.001/6.875
					b1–c1	0.001/3.059
60% (b)	170.4 (30.4) †	9921.8 (2367.3) †	697.58 (34.04) †	16 (15.9 ± 0.8)	a2–c2	0.001/1.813
					b2–c2	0.002/1.435
					a3–b3	0.001/8.243
80% (c)	89.2 (15.5) †	6867.1 (1577.5) †	213.91 (12.06) †	16 (16.1 ± 0.8)	a3–c3	0.001/19.602
					b3–c3	0.001/11.359

* Total work = repetition × sets × exercises, training volume = repetitions × sets × loads × exercises, RPE, rating of perceived exertion, † significant differences *p* < 0.05.

**Table 2:** Shows the variables of HRV and BP parameters.

Variables	Exercise Intensity	Pre-Exercise (1)	Post-Exercise (2)	10 Min. (3)	20 Min. (4)	30 Min. (5)	40 Min. (6)				
		Mean ± SD	Mean ± SD	Mean ± SD	Mean ± SD	Mean ± SD	Mean ± SD	Differences	∆%	(95% CI)	*p*/Cohen’s *d*
SBP	40% (a)	113.28 ± 8.9	125.35 ± 8.5	121.21 ± 8.7	117.57 ± 9.4	116.07 ± 11	114.64 ± 9.9	a1–a2 * b1–b2 *	10.65 ↑ 9.90 ↑	−20.1 to −4.1 −15.6 to −7.5	0.002/−1.342 0.001/−1.286
	60% (b)	116.85 ± 7.4	128.42 ± 8.1	124.07 ± 9.9	118.64 ± 8.3	119.07 ± 11.6	118.28 ± 8.2				
	80% (c)	117.71 ± 10.5	126.07 ± 8.6	124.21 ± 7.8	121.36 ± 8.8	120.21 ± 5.7	119.36 ± 9.2				
DBP	40% (a)	84.92 ± 10.5	74.78 ± 8.5	77.64 ± 8.3	79.14 ± 9.1	78.92 ± 8.5	83.28 ± 7.7				N/S
	60% (b)	85.5 ± 7.7	77.2 ± 7.1	79.4 ± 10.6	78.93 ± 11.2	81.42 ± 7.1	82.64 ± 7.9				
	80% (c)	80.71 ± 7.5	71.5 ± 12.1	76.35 ± 6.6	78.21 ± 9.5	77.92 ± 8.8	79.57 ± 6.6				
RMSSD	40% (a)	51.12 ± 17	16.80 ± 9.1	21.26 ± 9.6	24.39 ± 6.1	29.33 ± 6.5	36.17 ± 9.6	a1–a2 *	67.13 ↓	20.2 to 48.3	0.001/2.918
								a1–a3 *	58.41 ↓	14.8 to 44.8	0.001/2.539
								a1–a4 *	52.28 ↓	12.2 to 41.2	0.001/2.273
								a1–a5 *	42.62 ↓	6.1 to 37.4	0.004/1.853
	60% (b)	49.28 ± 19.5	13.58 ± 6.1	19.67 ± 7.8	24.01 ± 6.9	32.18 ± 8.6	35.64 ± 7.1	b1–b2 *	77.44 ↓	17.5 to 53.8	0.001/3.036
								b1–b3 *	60.08 ↓	14.4 to 44.8	0.001/2.519
								b1–b4 *	51.57 ↓	9.1 to 41.4	0.001/2.149
								c1–c2 *	70.51 ↓	12.3 to 62.8	0.002/3.196
								c1–c3 *	69.03 ↓	9.2 to 64.3	0.005/3.129
								c1–c4 *	62.00 ↓	5.2 to 61.1	0.015/2.809
								c1–c5 *	59.29 ↓	3.1 to 60.1	0.024/2.688
	80% (c)	53.29 ± 28.3	15.71 ± 5.3	16.50 ± 10.9	20.25 ± 8.9	21.69 ± 9.2	25.77 ± 9.5	a5–c5 ^$^	26.04	0.9 to 14.4	0.025/0.650
								b5–c5 ^$^	32.59	1.1 to 19.9	0.028/0.893
								a6–c6 ^$^	28.75	0.8 to 19.9	0.031/0.884
								b6–c6 ^$^	27.69	0.2 to 19.6	0.046/0.839
HF	40% (a)	46.33 ± 13.2	19.82 ± 6.1	18.9 ± 10.1	21.71 ± 6.5	25.07 ± 7.5	31.05 ± 6.9	a1–a2 *	57.21 ↓	10.8 to 42.1	0.001/2.292
								a1–a3 *	57.04 ↓	12.3 to 42.5	0.001/2.372
								a1–a4 *	53.14 ↓	10.8 to 38.4	0.001/2.129
								a1–a5 *	45.88 ↓	8.5 to 34	0.001/1.838
								a1–a6 *	32.98 ↓	2.7 to 27.8	0.012/1.321
	60% (b)	43.08 ± 16	19.48 ± 8.9	24.68 ± 16.1	24.74 ± 13.1	27.34 ± 11.5	31.61 ± 11.8	b1–b2 *	54.78 ↓	5.7 to 41.5	0.006/2.128
								b1–b3 *	42.71 ↓	0.4 to 36.3	0.042/1.592
								b1–b4 *	42.57 ↓	1.6 to 35.1	0.027/1.586
	80% (c)	48.59 ± 14.2	18.47 ± 8.2	19.41 ± 13.2	19.37 ± 12.6	21.69 ± 12	22.34 ± 12.4	c1–c2 *	61.98 ↓	16.9 to 43.3	0.001/2.604
								c1–c3 *	60.05 ↓	14.9 to 43.4	0.001/2.522
								c1–c4 *	60.13 ↓	14.2 to 44.2	0.001/2.527
								c1–c5 *	55.36 ↓	13.1 to 40.6	0.001/2.326
								c1–c6 *	52.02 ↓	10.8 to 41.6	0.001/2.270
								a6–c6 ^$^	28.05	0.2 to 17.2	0.045/0.753
LF	40% (a)	54.31 ± 13.9	80.85 ± 4.7	81.04 ± 10.2	78.15 ± 6.2	76.47 ± 7.8	75.95 ± 9.2	a1–a2 *	48.86 ↑	−40.5 to −12.5	0.001/−2.325
								a1–a3 *	49.21 ↑	−39 to −14.4	0.001/−2.342
								a1–a4 *	43.89 ↑	−35.8 to −11.8	0.001/−2.088
								a1–a5 *	40.80 ↑	−33.9 to −10.3	0.001/−1.941
								a1–a6 *	39.84 ↑	−33.2 to −10.1	0.001/−1.896
	60% (b)	52.9 ± 11.9	80.48 ± 8.9	75.16 ± 16.1	75.14 ± 13.2	72.58 ± 11.6	71.21 ± 13.1	b1–b2 *	52.13 ↑	−43.7 to −11.5	0.001/−2.417
								b1–b3 *	42.07 ↑	−43.9 to −0.6	0.041/−1.950
								b1–b4 *	42.04 ↑	−40.8 to −3.6	0.013/−1.949
								b1–b5 *	37.20 ↑	−38.1 to −1.3	0.030/−1.724
	80% (c)	51.98 ± 9.4	80.31 ± 10.7	79.74 ± 14.3	80.65 ± 12.5	77.36 ± 12.4	77.34 ± 12.6	c1–c2 *	54.50 ↑	−41.1 to −15.6	0.001/−2.483
								c1–c3 *	53.40 ↑	−42.8 to −12.7	0.001/−2.433
								c1–c4 *	55.15 ↑	−44.1 to −13.2	0.001/−2.513
								c1–c5 *	48.82 ↑	−41.4 to −9.3	0.001/−2.224
								c1–c6 *	48.78 ↑	−41.8 to −8.9	0.001/−2.222

* Different from pre-values; ^$^ different between sessions; SBP, systolic blood pressure; DBP, diastolic blood pressure; RMSSD, the root of the mean of the square of the difference in the RR intervals; HF, high frequency; LF, low frequency; N/S, not significant difference.

**Table 3:** Correlations between HRV metrics (RMSSD, HF, LF) and exercise variables (total repetition and volume -load).

Exercise Variables	Intensity	RMSSD Post		RMSSD 40 Min.		HF Post		HF 40 Min.		LF Post		LF 40 Min.	
		*r*	*p*	*r*	*p*	*r*	*p*	*r*	*p*	*r*	*p*	*r*	*p*
Total Repetition	40%	−0.431	0.124	0.121	0.680	−0.047	0.874	0.363	0.202	0.047	0.874	−0.365	0.199
	60%	−0.577 *	0.031	−0.235	0.420	−0.231	0.426	0.177	0.545	0.231	0.426	−0.177	0.546
	80%	−0.089	0.762	0.188	0.520	−0.156	0.593	0.324	0.259	−0.089	0.762	−0.324	0.258
Volume -load	40%	−0.221	0.447	0.007	0.981	0.124	0.674	0.269	0.353	−0.122	0.679	−0.271	0.350
	60%	−0.313	0.276	−0.093	0.753	0.054	0.854	0.214	0.462	−0.054	0.855	−0.214	0.462
	80%	0.238	0.413	0.107	0.715	0.249	0.390	0.371	0.191	0.238	0.413	−0.372	0.191

* significance at *p* < 0.05. RMSSD, root mean square of successive R-R intervals; HF, high frequency; LF, low frequency.

## Data Availability

Research data are available on request.
